# Case series of reports of pruritus and sipuleucel-T submitted to the Food and Drug Administration Adverse Event Reporting System

**DOI:** 10.1186/s40780-019-0156-0

**Published:** 2019-12-19

**Authors:** Graça M. Dores, Silvia Perez-Vilar, Manette T. Niu

**Affiliations:** 1 Division of Epidemiology, Office of Biostatistics and Epidemiology, Center for Biologics Evaluation and Research, U.S. Food and Drug Administration, 10903 New Hampshire Avenue, Silver Spring, MD 20993-0002 USA; 2 Office of Surveillance and Epidemiology, Center for Drug Evaluation and Research, U.S. Food and Drug Administration, 10903 New Hampshire Avenue, Silver Spring, MD 20993-0002 USA

**Keywords:** Sipuleucel-T, Provenge, Pruritus, Castration-resistant, Adverse events, FAERS

## Abstract

Sipuleucel-T, an autologous active cellular immunotherapy, is indicated for the treatment of asymptomatic or minimally symptomatic castration-resistant prostate cancer. The U.S. Food and Drug Administration Adverse Event Reporting System (FAERS) received a report of pruritus without rash following the second dose of sipuleucel-T in a patient who had otherwise not started any new medications concurrent with the first and second doses of sipuleucel-T. No further sipuleucel-T was administered, but symptoms persisted for at least 6 weeks despite treatment with several medications aimed at symptomatic relief of pruritus. Rash is the only dermatologic adverse event included in the sipuleucel-T U.S. package insert. A search of the FAERS database yielded seven additional U.S. reports of pruritus and sipuleucel-T identified as the primary suspect medication; two of these occurred prior to the administration of sipuleucel-T (following leukapheresis). In data mining analyses, pruritus following sipuleucel-T was not reported more frequently than expected when compared to all other adverse event-drug/biologic combinations in FAERS. Thus, pruritus following sipuleucel-T administration was rarely, but not disproportionately, reported to FAERS. Although we cannot exclude the possibility that diabetes, malignancy, or other conditions may have contributed to pruritus in our index patient, in view of the timing of sipuleucel-T therapy and onset of symptoms, a drug/biologic-related reaction is plausible. In the appropriate clinical scenario, sipuleucel-T (or its components) should not be overlooked as a potential etiological agent in pruritus.

## Background

On April 29, 2010, the U.S. Food and Drug Administration approved sipuleucel-T (Provenge, Dendreon Corporation), the first therapeutic cancer vaccine [1]. This autologous active cellular immunotherapy is indicated for the treatment of asymptomatic or minimally symptomatic metastatic castration-resistant prostate cancer [2]. Peripheral blood mononuclear cells are collected from the patient through leukapheresis; thereafter, antigen-presenting cells are harvested ex vivo, exposed to prostatic acid phosphatase fused to granulocyte-macrophage colony-stimulating factor (GM-CSF), and infused back into the patient [2]. In vivo, a T-cell-mediated immune response against prostate cancer cells expressing prostatic acid phosphatase is induced [2]. The leukapheresis and infusion process is repeated approximately every 2 weeks for a total of three doses.

## Main text

Health care providers, consumers, and manufacturers can submit reports of medication errors, adverse events (AEs), and product quality complaints to the Food and Drug Administration Adverse Event Reporting System (FAERS), a national spontaneous reporting system for drug and therapeutic biologics [3]. Medication errors and AEs are coded using terms in the Medical Dictionary for Regulatory Activities (MedDRA) [4]. FDA received a serious FAERS report of a 70-year-old male with a history of prostate cancer, hypertension, and diabetes who developed intense pruritus (with no apparent rash) involving the palms, head, torso, and soles of the feet 10 days after the second sipuleucel-T infusion. No other new medications were started before or during the time of receipt of the two sipuleucel-T doses. Treatment for pruritus included daily prednisone (60 mg), cetirizine (30 mg), doxepin (25 mg), and famotidine (40 mg) and as needed cyproheptadine (4 mg). The patient had not experienced symptomatic relief at the time the report was submitted, approximately 6 weeks after the last (second) sipuleucel-T infusion. Additional concomitant medications included dutasteride, flecainide, magnesium oxide, metformin, inhaled mometasone, montelukast, and olmesartan. Sipuleucel-T was discontinued after the second dose. According to the reporter (patient), his oncologist, allergist, and primary care physician attributed the pruritus to sipuleucel-T.

In a recent report of sipuleucel-T and AEs reported to FAERS through December 31, 2017, pruritus was not identified as one of the most commonly reported MedDRA Preferred Terms (PTs), nor was it disproportionately reported (i.e., more than twice expected, a cut-point commonly used by FDA to help assess if further investigation of the drug/biologic-event pair is needed) [5]. To determine if pruritus had been reported previously in association with sipuleucel-T, we searched FAERS for all U.S. reports from April 29, 2010 through December 31, 2018, with sipuleucel-T identified as the primary suspect medication (irrespective of timing of exposure and event) and containing a PT for one of the pruritus terms specified in Table 1. We conducted Empirical Bayesian data mining analyses using the Multi-Item Gamma Poisson Shrinker algorithm in the Oracle Empirica Signal System to assess for disproportionate reporting of pruritus following sipuleucel-T compared to all other AE-drug/biologic combinations reported to FAERS [6, 7].

**Table 1:** Reports of pruritus and sipuleucel-T submitted to the U.S. FDA Adverse Event Reporting System^a^

						Time to onset of symptoms from leukapheresis or infusion				
Case No.	Age (years)	Serious report? ^b^	Preferred Term^c^	Rash	Clinical description	Day No.	Leukapheresis No.	Infusion No.	Medical history^d^	Concomitant medications
Reports describing events in temporal association with sipuleucel-T										
1^e^	70	Yes	Pruritus generalized	No	Itching on palms, head, torso, and soles of feet	10	~	2	Hypertension, diabetes	Cetirizine, cyproheptadine, doxepin, dutasteride, famotidine, flecainide, magnesium oxide, metformin, mometasone (inhaled), montelukast, olmesartan, prednisone
2	69	No	Pruritus	Yes	Broke out in a rash, itching	2	~	2	NOS	Enzalutamide, metformin, tocopherol-fish oil
3	61	No	Pruritus	Yes	Tingling and discomfort in hands, then forearm rash (looked like red rings) with facial swelling; severe itching; eyelid swelling. Ten days after infusion was well except for itching	7	~	3	NOS	Aspirin, calcium, famotidine, lisinopril, vitamin D
4	81	Yes	Pruritus	Yes	Rash, itching, rigors, nausea, chest tightness, hypotension, vomited and hypoxic (85%) after 75–80% of infusion administered. Infusion discontinued. Admitted to hospital due to lack of response to medical therapy	0	~	1	Hypertension, hypercholesterolemia	Atenolol, calcium/cholecalciferol, citalopram, enalapril, simvastatin, vitamins
5	73	Yes	Pruritus	Yes	Swelling right upper arm (superficial thrombophlebitis), rash (IV catheter right hand), itching, tingling, cough, dyspnea	0	~	2	Deep vein thrombosis	NOS
Reports describing events in temporal association with leukapheresis (preceding sipuleucel-T)										
6	82	No	Injection site pruritus	No	Right arm injection site itching	1	1	~	Cardiac disorder, diabetes	Atenolol, furosemide, levothyroxine
7	63	No	Eye pruritus	No	Two separate events of allergic reaction with watery and itchy eyes associated with leukapheresis (tolerated three infusions without problem)	0	1 and 3	~	NOS	NOS
Report with insufficient information to assess temporal relationship with sipuleucel-T										
8	87	No	Catheter site pruritus	No	Catheter site pruritus; device related infection	NOS	NOS	NOS	NOS	Aspirin, calcium carbonate, denosumab, fiber, finasteride, hydrochlorothiazide/lisinopril, probiotic

Abbreviations: *IV* intravenous, *MedDRA* Medical Dictionary for Regulatory Activities, *NOS* not otherwise specified; ~ not applicable

^a^The FAERS database was searched for all U.S. reports of sipuleucel-T submitted between April 29, 2010 (FDA approval date) and December 31, 2018 (data-lock point, February 9, 2019). Among the 11 reports identified, three were excluded from this study (two duplicate reports and one report associated with enzalutamide therapy). All cases were reported among males. Two reports (Case no. 2 and 7) were premedicated prior to receiving sipuleucel-T (acetaminophen, diphenhydramine)

^b^A report is considered serious if the patient outcome results in death, life-threatening illness, hospitalization or prolongation of existing hospitalization, permanent disability, or birth defect (21 CFR Part 600.80. Post-marketing reporting of adverse experiences: https://www.ecfr.gov/cgi-bin/text-idx?SID=41533ceac3cc630045810ffe75304c63&mc=true&node=se21.7.600_180&rgn=div8)

^c^Pruritus-related MedDRA Preferred Terms: administration site pruritus, anal pruritus, application site pruritus, aquagenic pruritus, brachioradial pruritus, catheter site pruritus, cholestatic pruritus, ear pruritus, eye pruritus, eyelids pruritus, gingival pruritus, implant site pruritus, incision site pruritus, infusion site pruritus, injection site pruritus, instillation site pruritus, lip pruritus, medical device site pruritus, nasal pruritus, oral pruritus, pruritus, pruritus allergic, pruritus generalized, pruritus genital, senile pruritus, stoma site pruritus, tongue pruritus, tumor pruritus, uremic pruritus, vaccination site pruritus, vessel puncture site pruritus, vulvovaginal pruritus

^d^Other than prostate cancer

^e^Index case

We identified a total of eight unique reports submitted to FAERS with a pruritus-related PT and sipuleucel-T included as a primary suspect product between April 29, 2010 and December 31, 2018 (Table 1). In addition to the index case noted above, four reports described pruritus and rash occurring within 7 days of sipuleucel-T infusion; two reports described pruritus without rash occurring within 1 day following leukapheresis (prior to sipuleucel-T infusion); and one report did not provide sufficient details to enable temporal assessment. Our data mining analyses did not identify disproportionate reporting for sipuleucel-T and any of the pruritus-related PTs.

## Discussion

Rash is the only dermatologic AE included in the sipuleucel-T U.S. package insert (USPI) [8]. To our knowledge, pruritus following sipuleucel-T has not been previously reported in the literature. Pruritus may occur with or without rash. Systemic diseases (e.g., diabetes, hypothyroidism) may be associated with pruritus, and although pruritus is an uncommon paraneoplastic syndrome and is more typically associated with cancers of the lymphohematopoietic system, gastrointestinal and upper respiratory tracts, and (nonmelanoma) skin [9, 10], we cannot exclude the possibility that widespread malignancy, diabetes, or another systemic illness may have contributed to pruritus in the index patient. Medications, including GM-CSF, antihypertensives, statins, and others, have been reported to induce pruritus without skin changes [11, 12]. In addition, biologic agents, through their activation of the immune system, can incite cytokine-mediated hypersensitivity reactions, with manifestations overlapping with those of immediate, type I, antibody-mediated reactions or can be associated with delayed, type IV cutaneous hypersensitivity reactions that generally occur 7–21 days after exposure [13, 14].

The sipuleucel-T USPI describes acute infusion reactions and other signs and symptoms commonly associated with severe, type I hypersensitivity reactions (e.g., dyspnea, hypotension, tachycardia) [3, 15]. Generalized pruritus (with or without rash) is one of the dermatologic criteria for anaphylaxis and one of the potential symptoms of mucocutaneous, type IV hypersensitivity reactions [14, 15]. A prior FAERS-based sipuleucel-T case series did not identify pruritus as a potential safety signal [5]. However, that report did detect disproportionate reporting of acute infusion reactions, and PTs often associated with these reactions – nausea, hypoxia, tachycardia, presyncope – were also disproportionately reported [5]. Whether as a manifestation of an acute or delayed hypersensitivity reaction, pruritus has been plausibly associated with biologic agents [14] and is a labeled event for some myeloid growth factors [16]. While the role of leukapheresis in pruritus is uncertain, citrate used during leukapheresis may have a potential etiologic role.

FAERS is a large database that is particularly useful for identifying signals for rare AEs. However, it is a passive surveillance system with inherent limitations, including voluntary reporting, underreporting of AEs (e.g., AEs that are not reported, AEs that are not recognized to be associated with a drug/biologic), duplicate reporting, varying report quality with incomplete or missing information, variable reporting of product rechallenge/dechallenge information, and absence of denominator data [17, 18]. Therefore, although a causal association between a drug/biologic and an AE cannot be inferred from FAERS data, this unique resource can aid in the detection of rare or unexpected AEs that may merit further investigation.

## Conclusions

In this case series we describe eight reports of pruritus and sipuleucel-T as reported to FAERS over an 18-year period. Pruritus following sipuleucel-T administration was rarely, but not disproportionately, reported to FAERS. Widespread malignancy, diabetes, and other conditions may have contributed to pruritus in our index case, but in the context of timing of exposure to biologic therapy, particularly one possibly associated with GM-CSF [11, 12], and AE onset, a drug/biologic-related reaction is plausible. Pruritis may be debilitating among those affected, and in the appropriate clinical setting, sipuleucel-T or its components (e.g., residual GM-CSF) should not be overlooked as having a potentially relevant etiologic role.

## Data Availability

Refer to Main Text.

## Abbreviations

AE: Adverse event
FAERS: Food and Drug Administration Adverse Event Reporting System
GM-CSF: Granulocyte-macrophage colony-stimulating factor
MedDRA: Medical Dictionary for Regulatory Activities
Mg: Milligram
PT: Preferred Term
SOC: System Organ Class
U.S.: United States
USPI: Unites States Package Insert
